# Restorative and Endodontic Management of a Mandibular Canine With Two Roots and Two Canals: A Case Report

**DOI:** 10.7759/cureus.36460

**Published:** 2023-03-21

**Authors:** Mohammad D Aljanakh

**Affiliations:** 1 Department of Restorative Dentistry, College of Dentistry, University of Ha'il, Ha’il, SAU

**Keywords:** saudi, endodontics, root canal morphology, cone beam computed tomography, cbct, two roots, two canals, mandibular canine

## Abstract

The existence of a permanent mandibular canine with two roots and two canals is rare. This case report presents restorative and endodontic management of a mandibular canine with two roots and two canals of a 32-year-old Saudi woman with multiple periradicular lesions of mandibular anterior teeth. Cone beam computed tomography (CBCT) was used for accurate diagnosis of the root canal morphology. CBCT helped to accomplish the restorative and endodontic treatment in a conservative approach.

## Introduction

The main principle of endodontic treatment is to eliminate microorganisms from root canals and maintain this disinfected state [1]. To achieve this principle, the dental clinician needs to diagnose and perform chemomechanical debridement and obturation of root canals, which requires a thorough knowledge of root canal morphology [2]. Therefore, inadequate cleaning of the root canal system can lead to the failure of endodontic and related restorative treatments [3,4].

Most permanent mandibular canines (PMCs) have one root and one canal [5-9]. PMCs with two roots and two canals occur at a prevalence of 0.0 to 12.1% worldwide and 0.2 to 2.9% in Saudi Arabia [5,6]. Although this root canal morphology is rare, dental clinicians must be aware of variations in the number of roots and canals for proper case management, as unpredicted findings in root canal morphology can have a significant negative impact on endodontic treatment outcomes.

This paper reports a case of unilaterally occurring permanent mandibular canine (PMC) with two roots and two canals in a challenging management case. This is the first case of PMC with two roots to be reported from the northern region of Saudi Arabia.

## Case presentation

A 32-year-old Saudi woman presented to our private practice in Hail, Saudi Arabia, complaining of a history of pain and swellings related to the lower anterior region for the past four months. She explained that pain interfered with her daily life. She had no significant medical history. She had crowns on the upper and lower dental arches cemented for aesthetic reasons five years ago.

**Figure 1:**
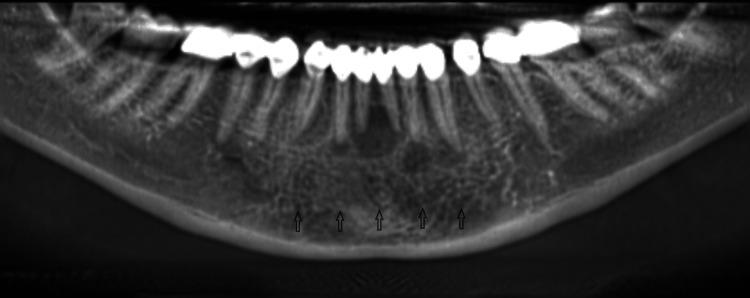
Orthopantomographic view of the mandibular jaw demonstrating multiple radiolucent lesions (arrows) and the difference in root length between the left and right PMCs. PMC: permanent mandibular canine

**Figure 2:**
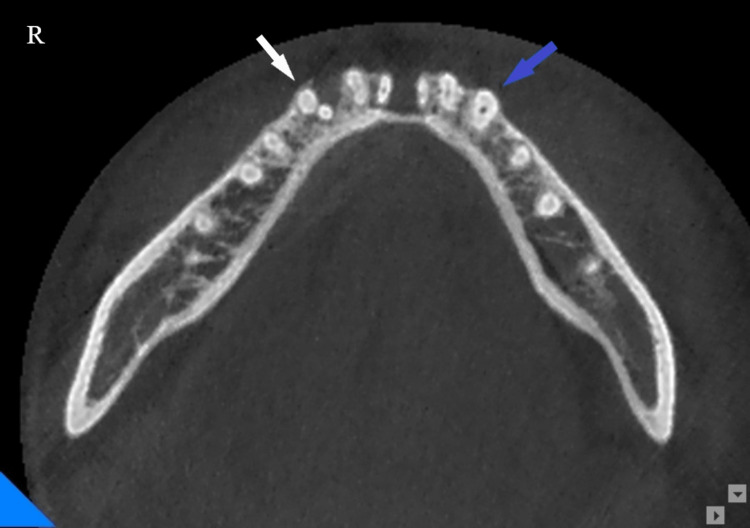
Cone beam computed tomography (CBCT), coronal view at the apical third level showing the two unilateral roots of the mandibular right canine (white arrow), and the single root mandibular left canine (blue arrow).

**Figure 3:**
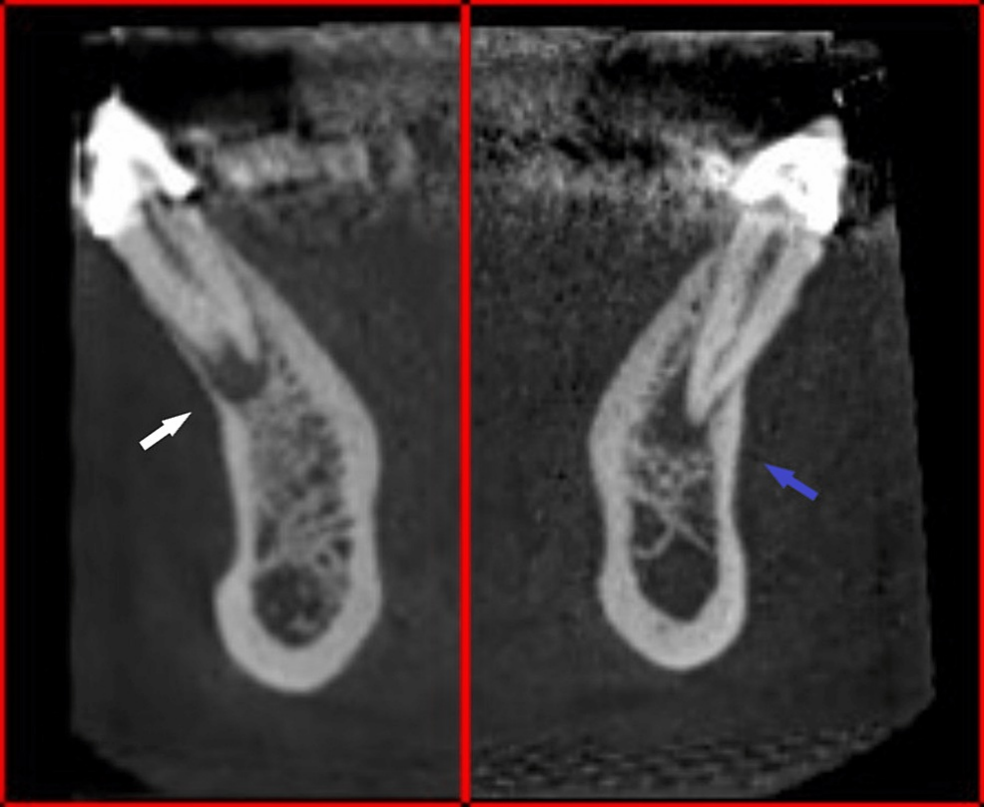
Cone beam computed tomography (CBCT) sagittal view showing root canal morphology of mandibular right canine (white arrow) and the single root mandibular left canine (blue arrow).

At the time of the examination, electric pulp tests and percussion vitality tests of the mandibular anterior teeth were not possible due to the presence of connected ceramic crowns from the lower right to the lower left canine. The patient refused to remove the zirconia crowns at that point. On the radiographic examination of the lower anterior teeth, multiple periradicular radiolucencies were detected (Figure 1). A cone beam computed tomography (CBCT) scan (MyRay, Imola, Italy) was performed to obtain detailed information about the teeth and to rule out suspected external root resorption of the right PMC (Figures 2, 3).

**Figure 4:**
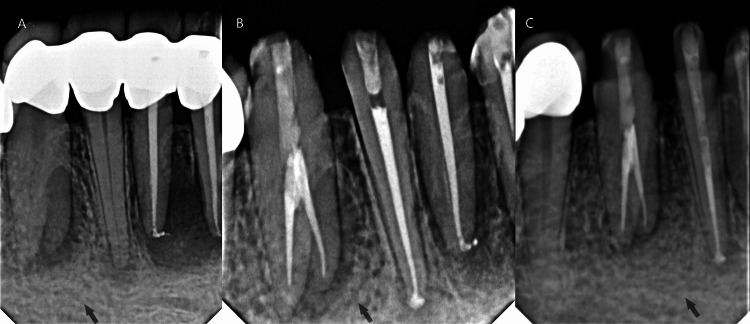
Periapical radiographs (PAs) of the right mandibular canine. Preoperative radiograph shows the canine root canal morphology and the related periradicular lesion (A); postoperative radiograph shows the obturation of the tooth and final restoration (B); follow-up radiograph 3 months after treatment shows initial healing (C).

The patient was informed that the connected crowns would be removed, and rest root canal treatments of the mandibular anterior teeth would be done before redoing the crowns. Informed consent was obtained prior to treatment. The crowns were then removed and vitality tests and percussion tests were performed. Based on CBCT, periapical radiographs, pulpal vitality tests, and percussion, the diagnosis was determined to be pulp necroses and chronic abscesses in the right PMC. Toot canal treatment of the right PMC was then completed. The CBCT and periapical radiographs of the right PMC showed that the buccal root was larger in diameter than the lingual root (Figure 2), and they were almost of the same length (Figures 3, 4), and the furcation was located at the apical root level (Figures 3, 4).

On a subsequent visit, the right PMC was accessed with a round diamond bur (No. 1014, Microdont, São Paulo, Brazil) and an Endo Z tapered safe-end bur (Dentsply/Maillefer, Ballaigues, Switzerland) with the help of a magnification loupes (3x Univet loupes, Rezzato, Italy). Anesthesia was administered, a rubber dam was applied, and the root canals were negotiated and instrumented manually to create a glide path using sizes 8 and 10 stainless steel K files M-Access (Dentsply Sirona, Ballaigues, Switzerland) and 10/.05 Glide Path File (Coltene Whaledent, Allstetten, Switzerland). A periapical (PA) radiograph was used to determine the working length and was confirmed with an apex locator (Root ZX mini; J. Morita Co., Kyoto, Japan). The canals were instrumented with a rotary system, HyFlex® EDM (Coltene Whaledent, Allstetten, Switzerland) using 20/.05 Preparation File and size 25/~ HyFlex OneFile, with 5.25% sodium hypochlorite irrigation. Canals were obturated using a hydraulic single cone technique with a matching gutta-percha size 25/~ HyFlex (Coltene Whaledent, Allstetten, Switzerland) using bioceramic sealer (CeraSeal, eta Biomed Co., Cheongju, Korea). The access cavity was then restored with a dual-cured composite (MultiCore Flow, Ivoclar Vivadent, Schaan, Liechtenstein) used with a dual-cure dental adhesive (Excite F DSC, Ivoclar Vivadent, Schaan, Liechtenstein) according to the manufacturer’s instructions (Figure 4).

**Figure 5:**
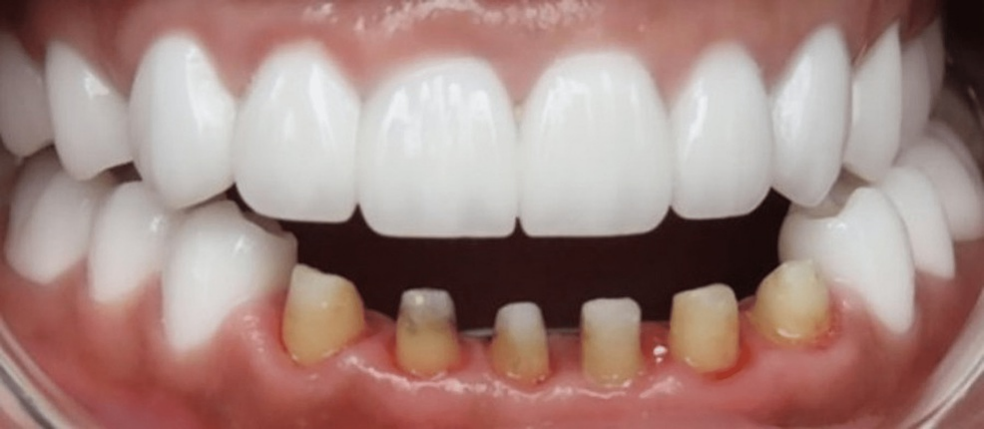
Prepared mandibular anterior teeth.

**Figure 6:**
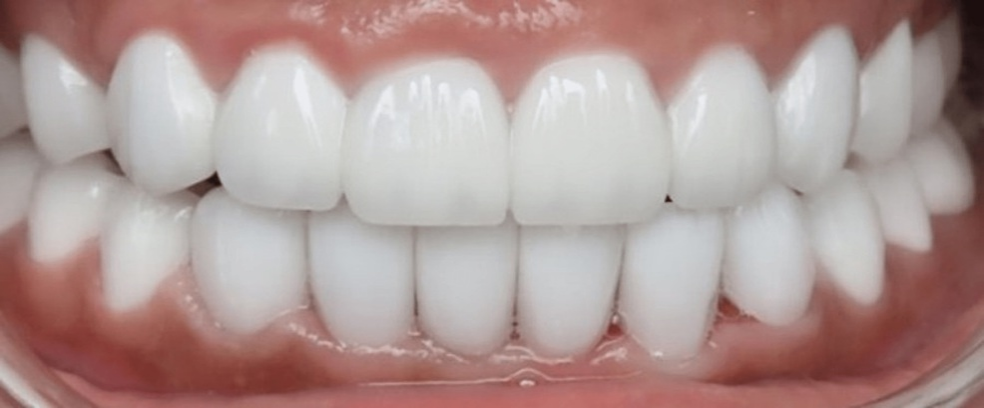
Zirconia crowns after temporary cementation.

Post-treatment follow-up after three months revealed initial clinical and radiological success. After performing root canal treatments for the anterior teeth, the treatment was completed as planned to restore the anterior teeth with zirconia crowns (Figures 5, 6).

## Discussion

PMCs are typically single-rooted, whereas double-rooted PMCs are rare compared to single-rooted PMCs [5-9]. Findings of the root canal morphology of PMCs in the Saudi population showed a rare occurrence of double-rooted PMCs ranging from 0.2% to 2.88% [7-9]. This paper reports the management of a PMC with two roots and two canals that occurred unilaterally in the dental arch. Although there have been a few cases of PMCs with two roots reported in Saudi Arabia, none have been reported from the northern region of the country [10-13].

The likelihood of two-root PMCs is uncommon, but when it occurs, it is mostly found unilaterally in the mandibular arch [14] and is found more often in women [15]. Moreover, the roots of unilateral PMCs with two roots are reportedly shorter than the roots of PMCs on the other side of the dental arch [15]. This is consistent with the details described in this case report.

The bifurcation level is another important variation that needs attention. According to Sharma et al. [16], the bifurcation root levels of PMCs with two roots were mostly at the mid-root. Instrumentation and obturation will be more challenging as the furcation levels occur more apically. In this case report, the bifurcation level was at the apical-root level (Figure 3) and the tooth crown was previously overprepared, which posed a challenge in accessing root canals using a conservative approach.

In certain clinical situations, CBCT has an invaluable advantage. Compared with conventional two-dimensional radiographs, CBCT is very useful to confirm a specific diagnosis and obtain accurate three-dimensional radiographic images [17]. it can be used to explore complex root canal morphology, allowing dental clinicians to preserve tooth structure [17]. However, the patient should not be exposed to higher doses of radiation except when needed, and their use should be for limited areas [17]. In this case report, CBCT was used as an aid in diagnosing multiple periapical lesions with complex root anatomy. Additionally, CBCT was used to rule out external root resorption of the right PMC.

Dental clinicians are responsible to reach the correct diagnosis and to provide proper treatment. For that, adequate exploration of root morphology is needed. Thus, performing an inappropriate endodontics treatment can be considered malpractice rather than a complication, which could lead to medicolegal consequences [18].

## Conclusions

Dental clinicians should not solely rely on common root canal morphology without considering anatomical variations. Proper knowledge of tooth root canal morphology besides other diagnostic auxiliary measures leads to the successful management of unexpected, uncommon cases. A mandibular canine with two roots is uncommon. Missing root canals in endodontics can result in treatment failure. Furthermore, CBCT radiography is a useful tool in cases where root and canal morphology is uncommon.
